# Ultrafast excited-state dynamics and fluorescence deactivation of near-infrared fluorescent proteins engineered from bacteriophytochromes

**DOI:** 10.1038/srep12840

**Published:** 2015-08-06

**Authors:** Jingyi Zhu, Daria M. Shcherbakova, Yusaku Hontani, Vladislav V. Verkhusha, John T. M. Kennis

**Affiliations:** 1 Biophysics Section, Department of Physics and Astronomy, Faculty of Sciences, VU University, De Boelelaan 1081, 1081 HV, Amsterdam, The Netherlands; 2 Department of Anatomy and Structural Biology, Albert Einstein College of Medicine Bronx, Bronx, New York 10461, USA; 3 Department of Biochemistry and Developmental Biology, Faculty of Medicine, University of Helsinki, Helsinki 00290, Finland

## Abstract

Near-infrared fluorescent proteins, iRFPs, are recently developed genetically encoded fluorescent probes for deep-tissue *in vivo* imaging. Their functions depend on the corresponding fluorescence efficiencies and electronic excited state properties. Here we report the electronic excited state deactivation dynamics of the most red-shifted iRFPs: iRFP702, iRFP713 and iRFP720. Complementary measurements by ultrafast broadband fluorescence and absorption spectroscopy show that single exponential decays of the excited state with 600 ~ 700 ps dominate in all three iRFPs, while photoinduced isomerization was completely inhibited. Significant kinetic isotope effects (KIE) were observed with a factor of ~1.8 in D_2_O, and are interpreted in terms of an excited-state proton transfer (ESPT) process that deactivates the excited state in competition with fluorescence and chromophore mobility. On this basis, new approaches for rational molecular engineering may be applied to iRFPs to improve their fluorescence.

In the past two decades, the advent of genetically encoded fluorescent proteins (FPs) of the GFP-like family has revolutionized the life sciences by enabling observation of living cells using fluorescence microscopy^1^,^2^,^3^. The next challenge is to visualize life processes in deep mammalian tissues and whole animals, where direct observation of cellular and organ function can make a key contribution to understanding of human health and disease. Fluorescence imaging in tissues poses a number of challenges, most prominently the high degree of absorption and scattering, which makes it difficult to obtain information through light apart from a thin upper layer. An ‘optical transparency window’ of mammalian tissues spans near-infrared (NIR) wavelengths of 650–900 nm, where hemoglobin, melanin and water absorption are minimal. NIR light can probe tissue at depths up to a couple of centimeters, enabling whole-body imaging of small mammals^4^,^5^.

However, even red-shifted FPs of the GFP-like family are not well suited for deep-tissue imaging: their excitation and emission maxima typically fall outside the optical transparency window. Considerable genetic engineering efforts to shift the GFP-like FPs to far-red resulted in their absorption shifted maximally to 611 nm^6^ and emission shifted to 675 nm^7^. The red-shift of absorption and emission of the GFP-like proteins is probably limited by the extent of the conjugated π-electron system^8^.

Recently, the new class of NIR FPs was developed from the subclass of phytochromes, called bacteriophytochromes (BphPs)^9^,^10^,^11^,^12^,^13^,^14^ Phytochromes constitute a family of light sensors in plants, fungi and bacteria^15^ ; their light-sensing module comprises, so-called PAS, GAF and PHY domains. The PAS-GAF fragment, which has a molecular weight of 35 kDa^16^, is sufficient for chromophore binding. Weak fluorescence of mutated phytochromes were first reported by Fischer and Lagarias^17^ and Vierstra and co-workers^18^. Phytochromes do not possess an autocatalytically formed chromophore as in the GFP-like proteins, but instead bind a bilin tetrapyrrole to a conserved cysteine residue in the PAS domain. In particular, BphPs bind biliverdin (BV) tetrapyrrole compound (Fig. 1a), which being a heme degradation product is a ubiquitous naturally occurring cofactor in mammalian tissues. iRFPs engineered from BphPs have their absorption and emission between 670 and 720 nm (Fig. 1b), both within the NIR optical transparency window. Fig. 1c shows a close up of the BV binding pocket in the *Rp*BphP2 X-ray structure^19^, the template protein from which iRFP713 and iRFP720 were derived, revealing the ZZZssa configuration of BV. Note that this structure provides only an approximate structural model for the iRFPs as various residues in the vicinity of BV were altered^9^,^14^.

Here, we aim to gain insights, through application of time-resolved laser spectroscopy, into the fluorescence function of NIR FPs, called iRFP702, iRFP713 and iRFP720^9^,^14^, which were recently derived from *R. palustris* BphPs, *Rp*BphP2 (iRFP713 and iRFP720) and *Rp*BphP6 (iRFP702), and are finding increasingly broad *in vivo* imaging application, including various cancer models^20^,^21^,^22^, parasitic disease^23^ and cardiac disease^24^ studies, stem cell approaches^24^,^25^ and transgenic animals^25^. Notably, the iRFP proteins efficiently utilize an endogenous BV chromophore of mammalian cells and tissues to become brightly fluorescent, which is in contrast to other reported NIR FPs that requires adding of an excess of external BV to acquire fluorescence^10^. Molecular knowledge of excited-state and reaction intermediate dynamics will contribute to a comprehensive picture of BphPs and iRFPs fluorescence function, and may serve as a basis for further rational engineering of advanced NIR FPs from BphPs.

## Results

### Ultrafast fluorescence and kinetic isotope effects

Figure 2 shows the results of a spectrally- and time-resolved fluorescence experiment on iRFP702 with excitation wavelength at 660 nm. Figure 2a (grey symbols) shows the kinetics at 702 nm in aqueous buffer. Global analysis indicates that the fluorescence decay can be described by a single exponential time constant of 749 ps (Fig. 2a, red line). Figure 2b are the same decay curves as in Fig. 2a but replotted on a logarithmic intensity scale. Figure 2c shows the decay-associated spectrum (DAS), which peaks at 702 nm and exhibits a vibronic shoulder near 760 nm. Experiments on iRFP713 and iRFP720 gave similar results, with single exponential decay time constants of 676 and 648 ps, respectively. Complete raw data and global analysis results are shown in Supplementary Information (SI3-Fig. S1). Time-resolved fluorescence experiments with iRFPs dissolved in D_2_O buffer showed a significant H/D effect on the fluorescence lifetime. In iRFP702, the fluorescence lifetime increased to 1.35 ns (Fig. 2a, open circles and blue line), corresponding to a kinetic isotope effect (KIE) of 1.8. Almost the same KIEs were obtained for iRFP713 and iRFP720 (SI3-Fig. S2 and Fig. S3). These observations are in line with those reported for wild type and point mutants of *Rp*BphP2 and *Rp*BphP3^26^.

### Ultrafast transient absorption

Figure 3 depicts the result of ultrafast transient absorption spectroscopy on iRFP702. In Fig. 3a, time resolved spectra were shown at different delay time after excitation with 660 nm laser pulse. We observe a simultaneous decay of the spectra throughout the whole band. Global analysis indicated that two time constants are required to adequately describe the spectral evolution, with time constants of 19 ps and 692 ps. The global fitting extracted spectra, which termed as evolution-associated difference spectra (EADS) are shown in Fig. 3b, together with a sequential decay kinetic model. Figure 3c shows kinetics at selected wavelengths along with the result from the global fit. The first EADS (black line) shows ground state bleach from 600–680 nm, excited-state absorption at 730 nm and stimulated emission at 780 nm. It evolves in 19 ps to the 2-nd EADS (red line), which only shows a small amplitude decrease, except in the stimulated emission region around 780 nm where a slight signal increase is observed. The latter observation implies that the 19 ps component is not associated with excited-state decay. We therefore assign the 19 ps component to a structural relaxation process in the excited state, in line with ps timescale processes observed earlier on other phytochromes^26^,^27^,^28^. The second EADS decays in 692 ps to baseline, which indicates that this component represents BV excited state decay. The time constant of 692 ps is similar to that observed in time-resolved fluorescence (749 ps, Fig. 2). Both EADS show an absorption band around 530 nm, which can be assigned to absorption from the (fluorescent) excited state to a higher excited state, as its decay lifetime is the same as the stimulated emission at 780 nm. No long-lived species are indicated in the global fit, in contrast to wild-type phytochromes where the 15Ea primary photoproduct Lumi-R is formed, indicating that the photocycle has been successfully abolished in the engineered iRFP proteins. The signal amplitude of any residual long-living species was too low for inclusion in the global analysis procedure; to further investigate this point, inspection of the raw ΔA spectra at 3 ns revealed a structure with an amplitude of about 2% of the initial signal, that might be interpreted as a photoproduct but is most likely a residual excited state (SI5-Fig. S6). Hence, we can put an upper limit of about 1% to photoproduct formation in iRFP702. H_2_O/D_2_O buffer exchange lead to longer excited-state lifetimes, in agreement with the streak camera results of Fig. 2.

Transient absorption experiments on iRFP713 and iRFP720 gave results similar to those of iRFP702, with a ps timescale relaxation in the excited state, a single exponential excited-state lifetime of 600–650 ps, an isotope effect on the lifetime (SI3-Fig. S4 and Fig. S5) and no appreciable photoproduct formation (SI5-Fig. S6). Time constants have been summarized in Table S1 (SI4-TS1) in the Supplementary Information. The only notable difference among the proteins is the extent and spectral location of excited-state absorption, which leads to different shapes and zero-crossings of the excited-state spectra.

## Discussion

**Figure 1:**
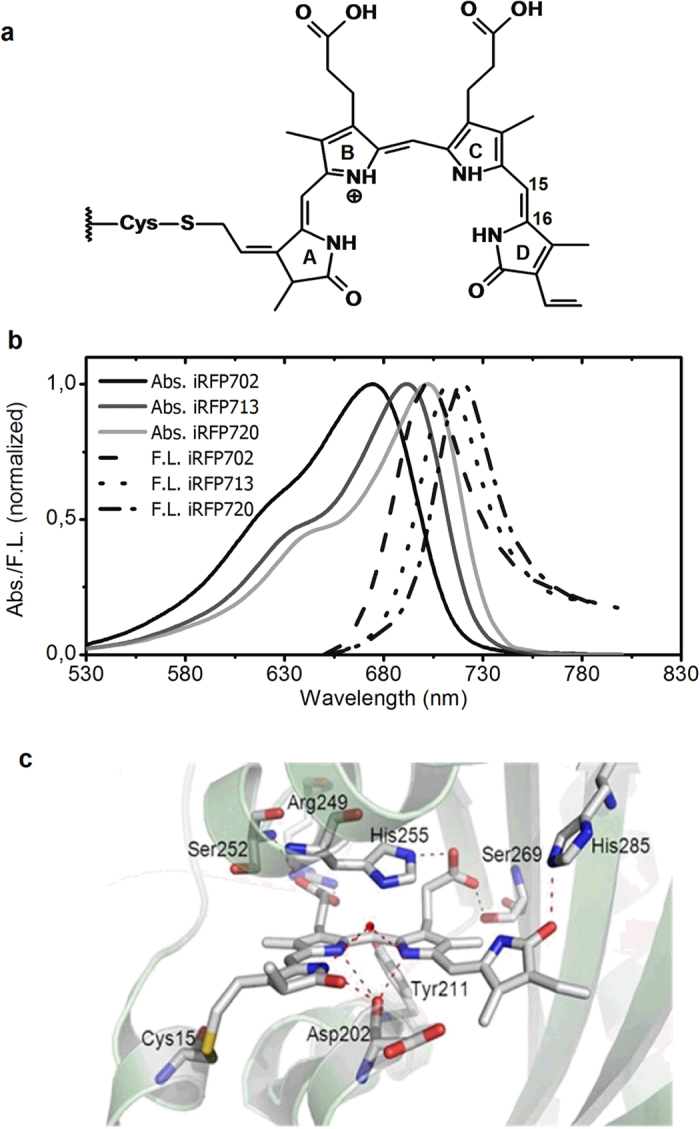
Properties of iRPF702, iRFP713 and iRFP720. (**a**) Biliverdin attachment to Cys in PAS domain of BphPs and these iRFPs; (**b**) Steady state absorption and emission spectra. (**c**) Close-up of the biliverdin chromophore binding pocket of *Rp*BphP2 (PDB code 4E04, ref. 19). Note that the model structure is only approximate.

**Figure 2:**
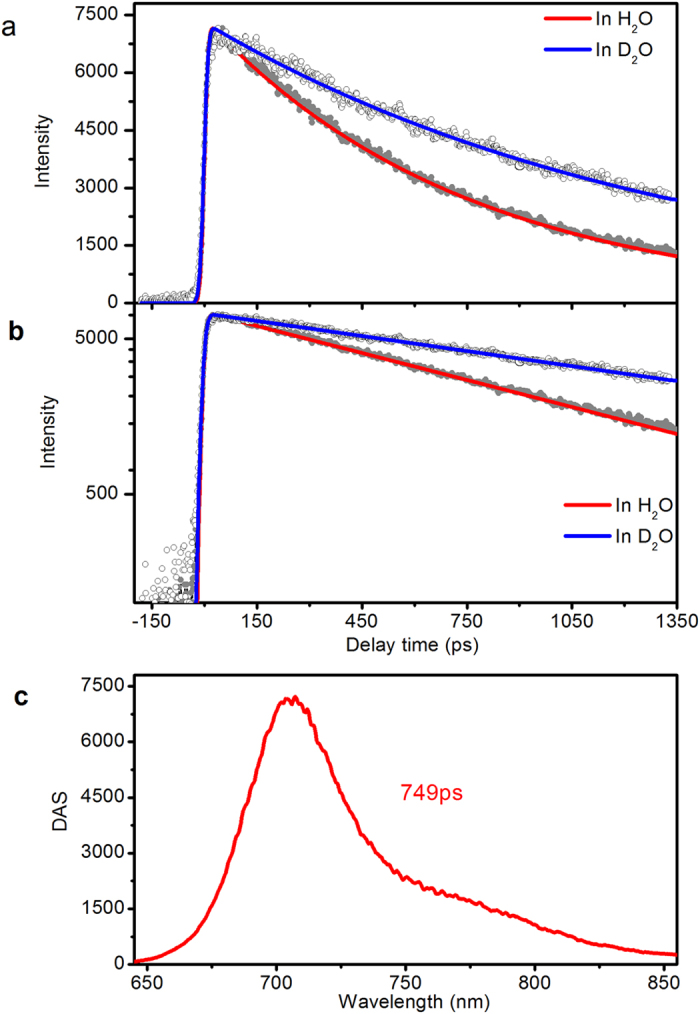
Time resolved fluorescence of iRFP702. (**a**) Decay of fluorescence at 702 nm in H_2_O and D_2_O with global fitted curves. (**b**) The same decay curves as in Fig. 2a but in logarithmic scale intensity. (**c**) DAS extracted by global fitting with a single exponential function. Data for iRFP713 and iRFP720 are shown in Fig. S3 and Fig. S2, respectively.

**Figure 3:**
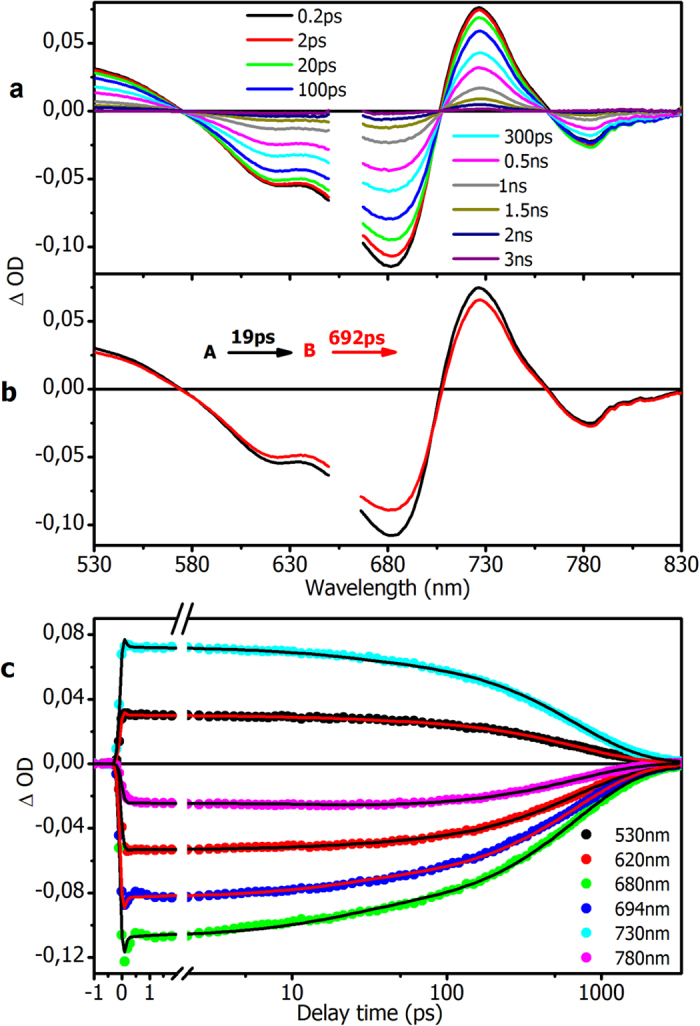
Transient absorption spectra and global analysis for iRFP702. (**a**) Time resolved spectra at different delay time; (**b**) EADS extracted by global fitting with a sequential decay kinetic model; (**c**) experimental data of time decay traces and global fitted ones. Data for iRFP713 and iRFP720 are in Figs S4 and S5, respectively.

Fluorescence efficiency is the most essential concern in the iRFPs designed for optical imaging. Such sub-ns lifetimes are consistent with their fluorescence quantum yields of 7–8%^14^ and indicate a BV inherent radiative lifetime of 9.5 ns, which is somewhat longer than that estimate of Toh *et al.* for *Rp*BphP3 (7.5 ns)^28^. The fluorescence quantum yield^14^ and lifetime reported here is close to a recently reported IFP1.4rev protein, which has slightly higher fluorescence quantum yield of ~8.7% possibly due to decreased BV structural heterogeneity^29^. The single exponential behavior indicates a rather homogeneous environment of BV, which contrasts with the commonly observed heterogeneous dynamics in BphPs and other phytochromes^26^,^28^,^30^,^31^. In addition, the single-exponential kinetics rule out that fast, tens of picoseconds excited-state decay components that are routinely observed in native phytochromes and are normally associated with Lumi-R formation^19^,^27^,^29^,^30^ contribute to fluorescence deactivation in iRFPs. These results agree with a recent resonant Raman study on iRFP713 which indicated a strongly reduced overall flexibility of the chromophore^32^. Generally, excited state BV relaxes through different energy dissipation channels: 1) NIR fluorescence, which makes iRFP suitable as probes for vivo imaging; 2) nonradiative decay to the ground state, possibly including excited state proton transfer^26^; 3) isomerization around the C_15 _= C_16_ double bond between pyrrole rings C and D of the bilin chromophore, resulting in formation of the Lumi-R primary intermediate^26^,^28^,^30^,^33^. The latter two processes compete with the useful fluorescence process and thus lower the fluorescence quantum yield if not suppressed. A previous study on fluorescence of phytochrome adducts with synthetic locked chromophores and mutants indicate that photoinduced isomerization provides a major route for excited state energy dissipation, and the fluorescence yield increased as isomerization is inhibited^34^. For all three iRFPs studied here, no significant photoisomerization species are observed, which implies that the natively occurring ring D isomerization about the C_15 _= C_16_ bond (Fig. 1a) has been efficiently blocked in all three iRFPs. A rigid biding site environment was formed and thus a brightness of the fluorescence is realized in the studied iRFPs.

A key observation is the H_2_O/D_2_O kinetic isotope effect (KIE) on the fluorescence lifetime of all three iRFPs, which may imply that a proton-motion dependent excited-state deactivation process takes place, just as previously observed in the wild-type *Rp*BphP2 and *Rp*BphP3 and their point mutants^26^,^28^. In that work, the KIE was interpreted to arise from an ESPT process that occurs at the pyrrole nitrogens of rings A, B or C (Fig. 1a) to the coordinated backbone carbonyl of a conserved Asp residue in PASDIP motif in GAF domain of BphPs, or to a bound pyrrole water molecule, and deactivates the BV excited state. Thus, the proposed ESPT forms a competing reaction with fluorescence. Recent fluorescence line narrowing experiment at cryogenic temperatures (6 K) demonstrated that the BV excited state is indeed coupled to proton motion, which was enhanced when altering the environment around the pyrrole rings^35^.

**Figure 4:**
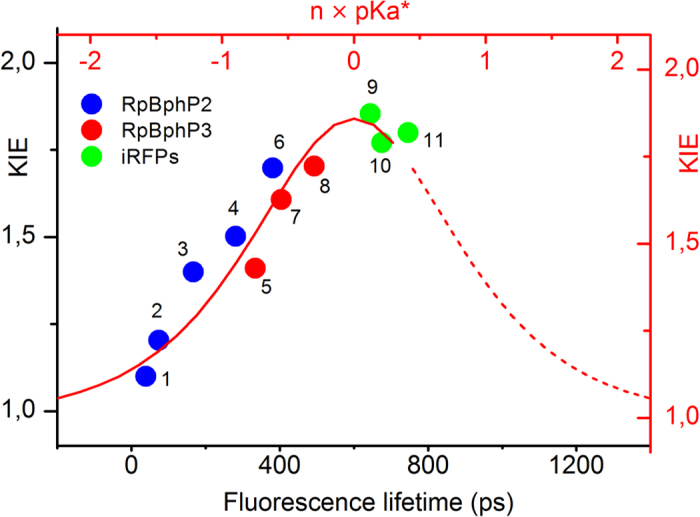
KIEs of iRFPs, *Rp*BphP2 and *Rp*BphP3 versus their fluorescence lifetimes. Dots represent current experimental results and from refs 26 and 28. The red curve is calculated according to Marcus BEBO expression model. The constant n before pKa* is a random positive scale factor. Number 1 to 11 represent life time from protein (1) WT PAS-GAF-PHY (short phase), (2) WT PAS-GAF (short phase), (3) WT PAS-GAF-PHY, (4) WT PAS-GAF, (5) WT PAS-GAF-PHY, (6) WT PAS-GAF-PHY/D202A, (7) WT PAS-GAF, (8) WT PAS-GAF-PHY/D216A, (9) iRFP720, (10) iRFP713 and (11) iRFP702.

Interestingly, there appears to be a correlation between fluorescence lifetime and KIE in natural BphPs and BphP-engineered iRFPs, as shown in Fig. 4 where current and published^26^,^28^ data are summarized. Note that the lifetimes of wild type *Rp*BphP2 appear twice because of their bi-exponential decays. We observe that for short lifetimes (<100 ps), the KIE is close to 1, while for increasing lifetimes the KIE goes up to 1.8. This observation indicates that the proposed ESPT process becomes more important when the fluorescence lifetime becomes longer.

The observed lifetime dependence of the KIE may be interpreted in two ways:

(i) There is a competition between a fast, proton transfer *independent* deactivation and an ESPT process that has a relatively slow rate (in the order of ns) and fixed KIE. The former process most probably corresponds to ring D rotation followed by internal conversion either to Pr or to Lumi-R, or mobile groups elsewhere on the chromophore. As a consequence, in case of short lifetimes such as the 45 ps component in *Rp*BphP2^26^ ESPT is not important and the KIE is close to unity. In case of long lifetimes, ring D rotation is inhibited, ESPT becomes important and a significant KIE is observed. The latter situation applies to iRFPs, *Rp*BphP3 and the long lifetime components of *Rp*BphP2^26^,^28^.

(ii) For all observed lifetimes in *Rp*BphP2, *Rp*BphP3 and iRFPs, ESPT is the leading cause of excited-state deactivation. Here, in wild type *Rp*BphP2 ESPT is faster than in the iRFPs and in *Rp*BphP3. This may be correlated with a larger twisting of ring D in wild type *Rp*BphP2, which could increase the acidity at the pyrrole rings in the excited state^26^. In iRFPs and *Rp*BphP3, ring D is more restrained, twists less and the pyrrole rings become less acidic in the excited state with ensuing slower ESPT.

To investigate possibility (ii), we described the KIEs and ESPT dynamics by a Marcus bond-energy bond-order (BEBO) expression^36^,^37^ of two state proton transfer model, as the calculated bell like curve in Fig. 4. Details of this calculation are described in SI2. Since the information of free energy of activation is lacking, the excited state pKa* is scaled by a factor n. The result shows that all BphP proteins considered here are located at the low and negative going pKa side of the bell-like curve, with the current sample iRFP702, iRFP713 and iRFP720 located near the top at pKa* = 0. Note that because the experimental data do not cover the entire range of the bell-like curve, the peak position could not be well determined. Yet, the observed trend can be roughly described by the Marcus BEBO model in the left side of the bell-like curve, which results in negative pKa* values of the BV chromophore, implying that BV would be a strong photoacid. In canonical phytochromes, the ground state pKa for chromophore deprotonation is higher than 9^38^, so it would be lowered 9 units in the iRFPs’ excited state, and even more so in wild type *Rp*BphP2 and *Rp*BphP3. This situation would be similar to that of GFP, where the chomophore pKa drops 9 units in the excited state and ESPT ensues with a time constant of 3 ps^39^. However, BV is not expected to be a strong photoacid^40^, and accordingly ESPT, if it occurs, proceeds 10^2^–10^3^ times slower. To fully understand the data trend and bring it into theoretical model description, more experimental data from different iRFPs are needed. At this point we consider possibility (i) most likely, and we provisionally discard possibility (ii) for the origin of lifetime dependence of ESPT in *Rp*BphPs and these three iRFPs.

We stress that although clear KIEs are observed for the iRFP fluorescence lifetime, no transient (de)protonated species are observed in the transient absorption data. We therefore explore additional mechanisms that might account for the observed isotope effects. An alternative explanation for the observed H_2_O/D_2_O KIE effects rests in the energy gap law for radiationless transitions, which predicts that internal conversion (IC) in large molecules is mediated primarily by high-frequency C-H and N-H stretch modes^41^. In porphyrins and bilins, deuteration of exchangeable protons may lead to a decreased Franck-Condon overlap of N-D accepting modes of the pyrrole rings and hence in a decreased IC rate. Indeed, in the past small H/D KIEs of 1.2 on fluorescence lifetimes were observed in free-base porphyrins, with lifetimes of 12.5 ns for protonated and 15 ns for deuterated molecules at the imino hydrogens^42^. However, such explanation is unlikely to apply for iRFPs and BphPs because the IC rate constants in question are at a least order of magnitude smaller than the fluorescence decay rates in iRFPs, and hence play no appreciable role in their fluorescence deactivation. We therefore prefer a model where ESPT partly contributes to excited-state deactivation, where the (de)protonated species are so short-lived that they escape detection.

In summary, by using ultrafast time-resolved spectroscopy, we found that fluorescence lifetimes became longer and, thus, the quantum yield are higher in the recently designed iRFP702, iRFP713 and iRFP720 NIR FPs. Single exponential decay lifetimes and lack of photoproducts states indicate that the natively occurring ring D isomerization about the C_15 _= C_16_ bond has been efficiently blocked. Using isotope studies, we have found that a proton-motion dependent process, possibly ESPT, constitutes a significant cause of excited-state deactivation that diminishes fluorescence in iRFP702, iRFP713 and iRFP720. Future design strategy to improve the fluorescence quantum yield of the BphP-based NIR FPs will have to address minimization of this process in addition to further restricting the mobility of the chromophore.

## Methods

### Protein samples expression and purification

The iRFP702, iRFP713 and iRFP720 genes were cloned in the pBAD/His-B vector (Invitrogen)^14^. The iRFP702, iRFP713 and iRFP720 proteins with polyhistidine tags on the N-termini were expressed in LMG194 bacterial cells (Invitrogen) bearing the pWA23h plasmid encoding heme oxygenase under the rhamnose promoter^13^,^14^. To initiate protein expression, bacterial cells were grown in RM medium supplemented with ampicillin, kanamycin and 0.02% rhamnose for 5 h at 37 °C. Then 0.002% arabinose was added and bacterial culture was incubated for additional 12 h at 37 °C following by 24 h at 18 °C. Proteins were purified using Ni-NTA agarose (Qiagen). Ni-NTA elution buffer contained no imidazole and 100 mM EDTA. The elution buffer was substituted with PBS buffer using PD-10 desalting columns (GE Healthcare).

### Ultrafast fluorescence measurements

Time-resolved fluorescence experiments were performed with the setup described earlier^28^,^43^. All the samples were excited with ~200 fs pulses at 660 nm delivered from an optical parametric amplifier (Coherent OPA), which was pumped by an integrated Ti-sapphire laser system operating at 100 kHz. The excitation pulses were focused on a 1 cm path length quartz cuvette placed on a magnetic stirrer. Samples’ concentrations were measured with their corresponding absorbance of ~0.15 O.D. Fluorescence was collected at right angle to the excitation using achromatic lenses and detected through a sheet polarizer set at magic angle with a Hamamatsu C5680 synchroscan streak camera and a Chromex 250IS spectrograph. The dispersed light was converted to electrons at the photocathode and time-resolved by varying the voltage applied to sweep electrodes. A microchannel-plate (MCP) was used to amplify the photocathode signal and projected to a phosphor screen where it was visualized by a CCD camera. Time resolution was estimated by the instrument responding of around 22 ps.

### Ultrafast absorption measurements

Transient absorption spectroscopy was carried out as described in previous reports^28^,^44^. A 30 fs laser system (Legend, Coherent) with 1 kHz repetition rate pumps a optical parametric amplifier (Coherent TOPAS) to generate 660 nm for excitation. Excitation pulse with energy of ~80 nJ/pulse was sent to an optical delay line for timing control, After the delay line, the pulse was chopped every other pulse with a 500 Hz phase stabilized optical chopper. Broad band probing white light was generated by focusing part of fundamental output of the laser (800 nm) to a laterally rotating 2 mm-thickness CaF_2_ plate. The polarization of the probe light was at the magic angle with respect to the excitation. Cross correlation of the pump and probe pulse is around ~120–150 fs throughout the whole probing wavelength. The samples were held in a mechanically shaken cuvette cell with 1 mm optical path, ~0.4 O.D. of all samples was used.

### Data Analysis

Transient absorption and time resolved fluorescence data were analyzed with model-based global fitting programed in LabView^45^,^46^. Details of global fitting procedure are incudued in Supplementary information material (SI1).

## Additional Information

**How to cite this article**: Zhu, J. *et al.* Ultrafast excited-state dynamics and fluorescence deactivation of near-infrared fluorescent proteins engineered from bacteriophytochromes. *Sci. Rep.* **5**, 12840; doi: 10.1038/srep12840 (2015).

## Supplementary Material

Supplementary Information
