# Supplementary material for: Ultrafast excited-state dynamics and fluorescence deactivation of near-infrared fluorescent proteins engineered from bacteriophytochromes
Source: Sci Rep. 2015 Aug 6;5:12840. doi: 10.1038/srep12840 (PMC4526943; doi:10.1038/srep12840)
Supplement: Supplementary Information [file srep12840-s1.pdf]

Ultrafast excited-state dynamics and fluorescence deactivation of near-infrared fluorescent proteins engineered from bacteriophytochromes

Jingyi Zhu,[1] Daria M. Shcherbakova [2] Yusaku Hontani,[1] Vladislav V. Verkhusha [2, 3] and John T.M. Kennis*,[1]

[1] Biophysics Section, Department of Physics and Astronomy, Faculty of Sciences, Vrije Universiteit, De Boelelaan 1081, 1081 HV, Amsterdam, The Netherlands

[2] Department of Anatomy and Structural Biology, Albert Einstein College of Medicine Bronx, Bronx, New York 10461, USA

[3] Department of Biochemistry and Developmental Biology, Faculty of Medicine, University of Helsinki, Helsinki 00290, Finland

[*] Email: j.t.m.kennis@vu.nl

Supplementary Information

SI1: Kinetic model global analysis

SI2: Marcus BOBE calculation

SI3: Supported Fig.S1---Fig.S5

SI4: Supported table: TS1

SI5: Supported Fig. S6

SI6: References

SI1: Kinetic model global analysis

Transient absorption and time resolved fluorescence data were analyzed with model-based global fitting program implemented in LabView[1,2] Global target analysis can provide an integral and more compact description of the system dynamics than a single wavelength time trace fitting. The physics and mathematics basis for global target analysis of the measured data matrix ΔA (λ, t) is that the variable delay time t and wavelength λ are independent. That is, the measured ΔA (λ, t) is a superposition of several species components and can be separated as:

$$\Delta A(\lambda,t)=\sum_{i=1}^{N} C_i(t)\Delta\varepsilon_i(\lambda)$$

....(1)

where $C_i(t)$ and $\Delta\varepsilon_i(\lambda)$ are the concentration and extinction coefficient of the generalized species component Ni respectively. A parametric dependent kinetic model based on the first order reaction was resorted to $C_i(t)$ and by globally fitting the data ΔA (λ, t) to extract the spectral components of $\Delta\varepsilon_i(\lambda)$, which generally is the species extinction coefficient and has different nomination according the specific kinetic models used. Based on the kinetic model, $C_i(t)$ was constructed and resolved by the differential equation:

$$\frac{dC_i(t)}{dt}=-k_iC_i(t)+\sum_{j\neq i}\chi_{ji}k_jC_j(t)$$

....(2)

Where k_i represent the total decay of state species i and χ_{ji} is the branching ratio of species j to i. The reconstructed $C_i(t)$ were convoluted with the instrument response function (IRF, normally a Gaussian shape) to globally fit the data by minimizing:

$$\sqrt{\left(\Delta A(\lambda,t)-\sum_{i=1}^{N} C_i(t)\Delta\varepsilon_i(\lambda)\right)^2}$$

....(3)

The initial guess for $\Delta\varepsilon_i(\lambda)$ were given by multiplying the pseudoinverse of matrix $C_i(t)$ with matrix ΔA (λ, t). The fitting leads to a set of rate constants and branch ratios and the corresponding species spectra $\Delta\varepsilon_i(\lambda)$ simultaneously. This global fitting process

was realized by using genetic evolution method to searching the minimum value of (3), a program developed by the author using computer language LabView.

In order to extract species spectra $\Delta\varepsilon_i(\lambda)$, a specific kinetic reaction model has to be proposed. In general, two simple standard kinetic reaction model can be assumed for simply describing the experimental data, as shown in scheme 1) and 2) below. In scheme 1), each species decay independently after photo-excitation, which results in species spectra called "Decay Associated Difference Spectra (DADS)". In scheme 2), species decays sequentially from one to another after the excitation create the first species, this model extract species spectra as "Evolution Associated Difference Spectra (EADS)". Real situation could be complicated which includes mixing of both above cases, a specific kinetic model such as in scheme 3) whose extracted species spectra was called " Species Associated Different Spectra (SADS)". In our studied samples here, dynamics very simple, DADS or EADS model are sufficient to describe our data.

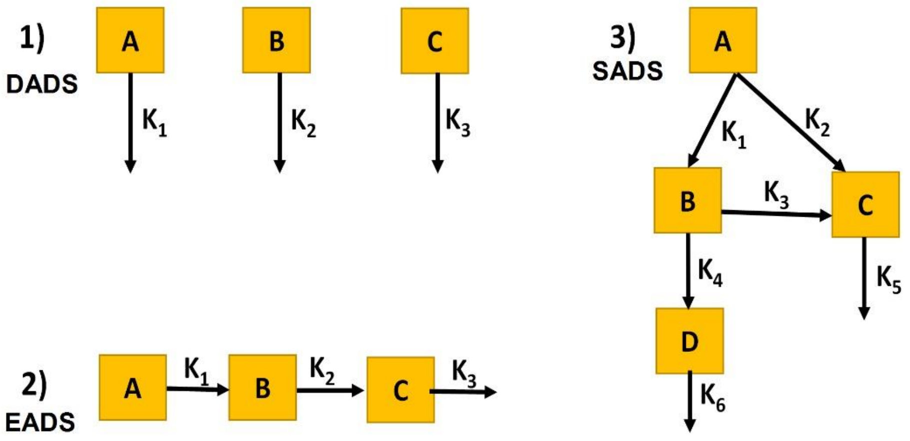

SI2: Marcus BOBE calculation

Marcus Bond-Energy-Bond-Order model for calculation KIE of the ESPT can be found in previous reports.[3,4] Basically the KIE of proton transfer k^H/k^D can be described by a formula:

$$\frac{k^H}{k^D} = \exp^{A\{1-[x*\tanh(x)-\ln\cosh(x)]/\ln 2\}} \qquad \ldots\ldots\ldots\ldots\ldots\ldots\ldots\ldots (4)$$

Where A is amplitude constant, the variable x is expressed as:

$x = \frac{\Delta G^0 \ln 2}{2\Delta G_0^{\#}}$ .... (5)

$\Delta G_0^{\#}$ is the intrinsic activation energy of proton transfer and is a constant, while ΔG^0 is related to excited state pKa* and temperature as:

ΔG^0 = -RTlnKa*=RTpKa*/ln(e).. .... (6)

Thus at room temperature, the variable x in (4) can be expressed as:

$x = npKa^*$ .... (7)

n is a random positive scale constant for pKa*. Ka* is the excited state proton dissociation equilibrium constant. Use formula (a) a KIE curve can be calculated with scaled pKa*, as shown in figure 4 of main manuscript.

SI3: Supported Fig.S1---Fig.S5

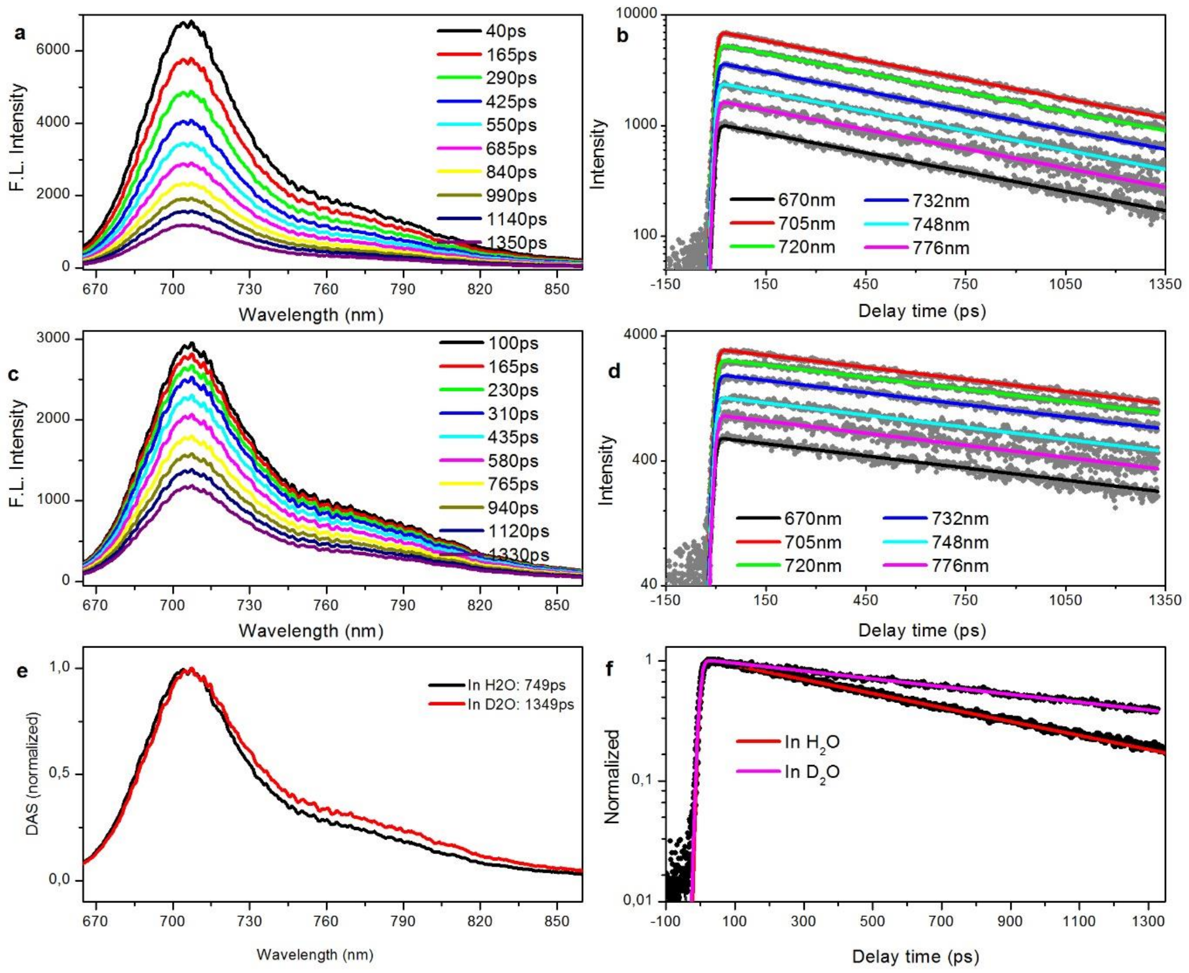

Figure S1. Time resolved fluorescence results of iRFP702 in H_2O and D_2O. (a) Time resolved fluorescence spectra in H_2O; (b) Decay traces and global fitted ones in H_2O; (c) Time resolved fluorescence spectra in D_2O; (d) Decay traces and global fitted ones in D_2O; (e) Global fitting extracted DAS in H_2O and D_2O; (f) comparing of the fluorescence lifetimes in H_2O and D_2O at wavelength around 720nm.

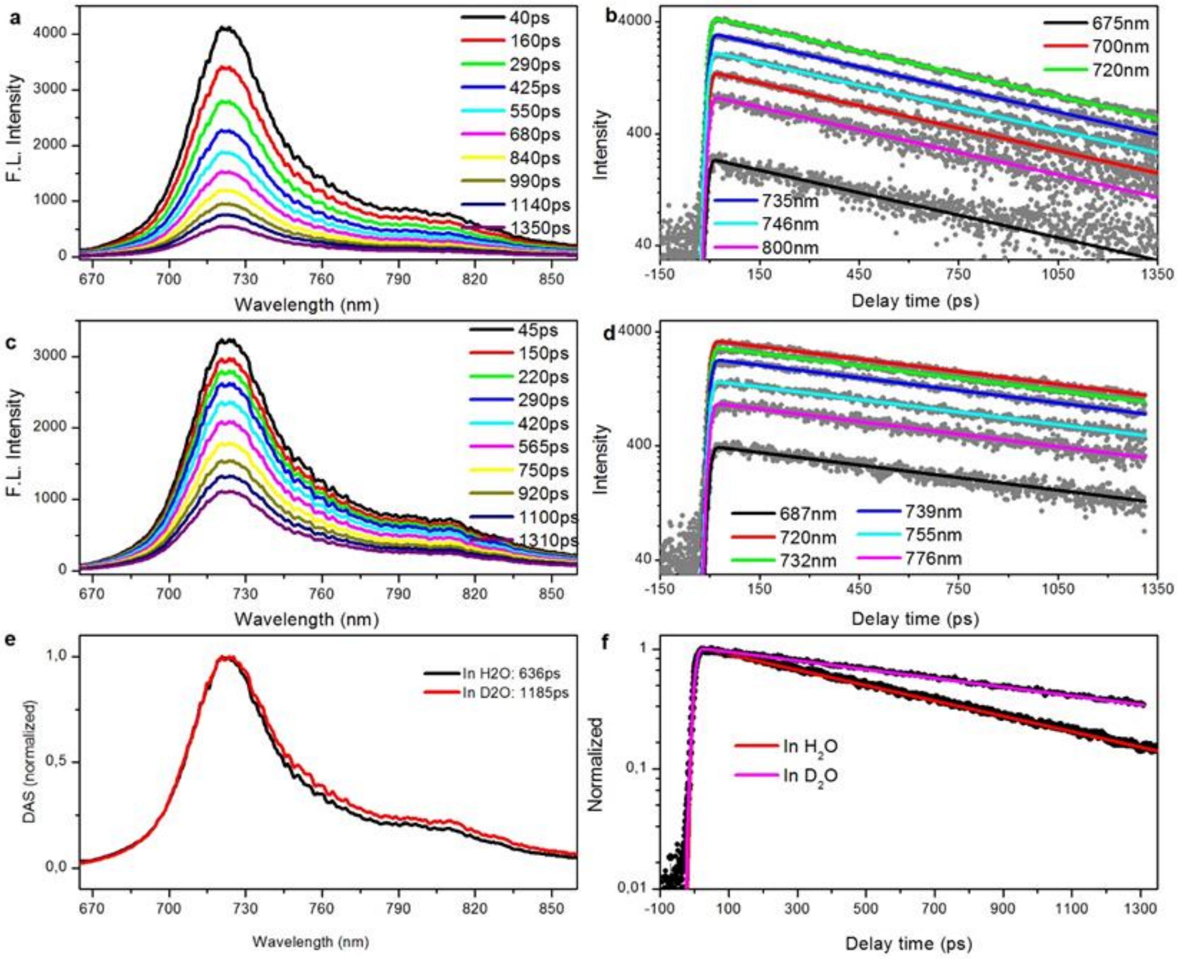

Figure S2. Time resolved fluorescence results of iRFP720 in H_2O and D_2O. (a) Time resolved fluorescence spectra in H_2O; (b) Decay traces and global fitted ones in H_2O; (c) Time resolved fluorescence spectra in D_2O; (d) Decay traces and global fitted ones in D_2O; (e) Global fitting extracted DAS in H_2O and D_2O; (f) comparing of the fluorescence lifetimes in H_2O and D_2O at wavelength around 725nm.

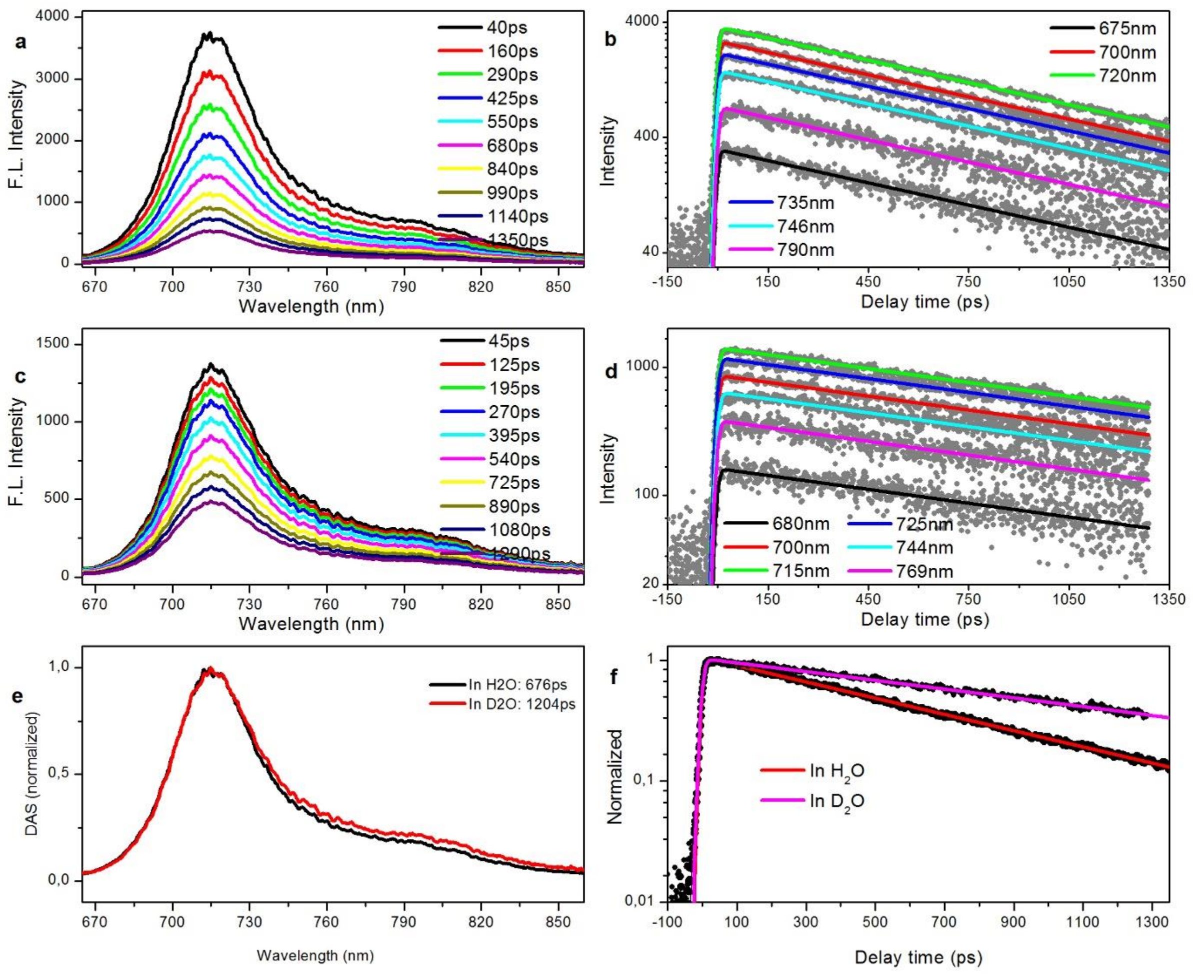

Figure S3. Time resolved fluorescence results of iRFP713 in H_2O and D_2O. (a) Time resolved fluorescence spectra in H_2O; (b) Decay traces and global fitted ones in H_2O; (c) Time resolved fluorescence spectra in D_2O; (d) Decay traces and global fitted ones in D_2O; (e) Global fitting extracted DAS in H_2O and D_2O; (f) comparing of the fluorescence lifetimes in H_2O and D_2O at wavelength around 720nm.

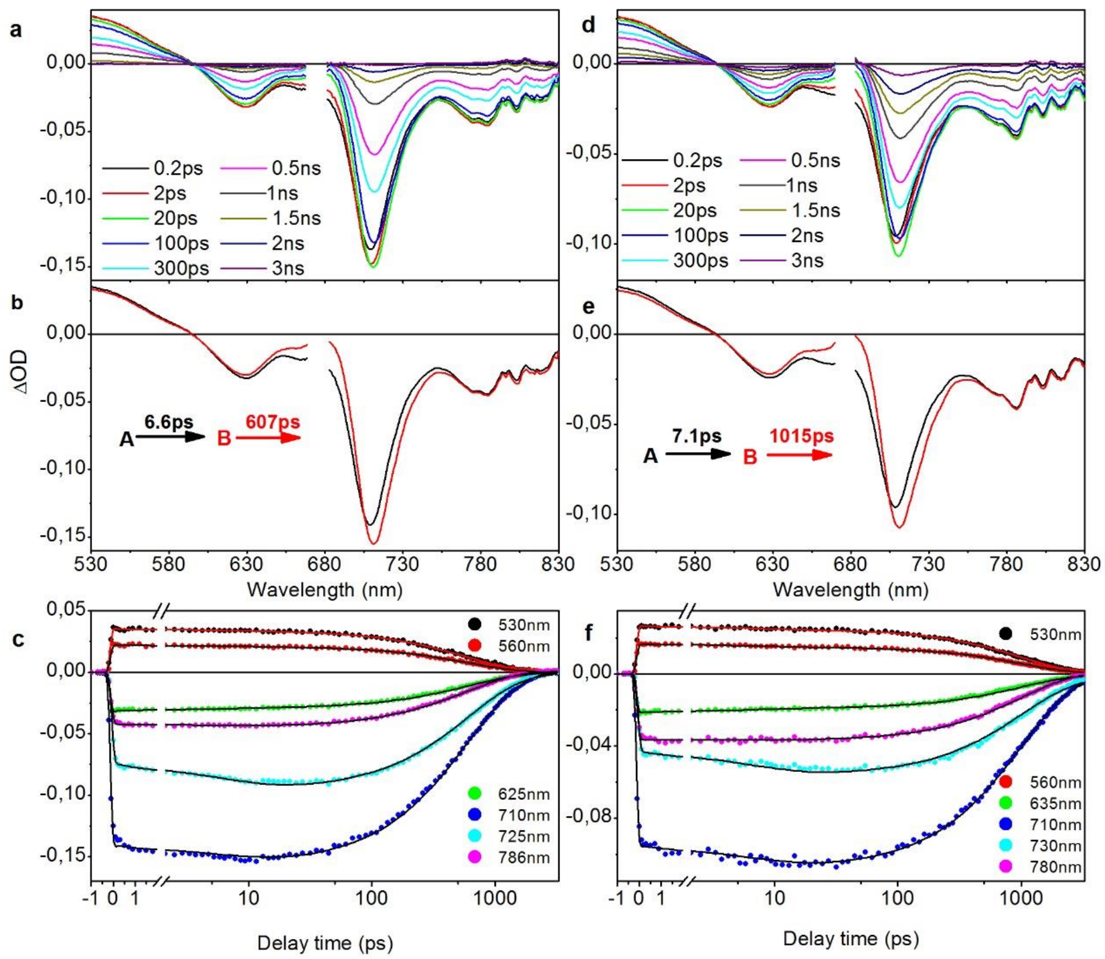

Figure S4. Transient absorption experimental results of iRFP713 and analyzed data in H_2O and D_2O. (a) Time resolved spectra in H_2O; (b) Global fitted EADS in H_2O; (c) Time decay traces in different wavelength and global fitted ones in H_2O; (d) Time resolved spectra in D_2O; (e) Global fitted EADS in D_2O; (f) Time decay traces in different wavelength and global fitted ones in D_2O.

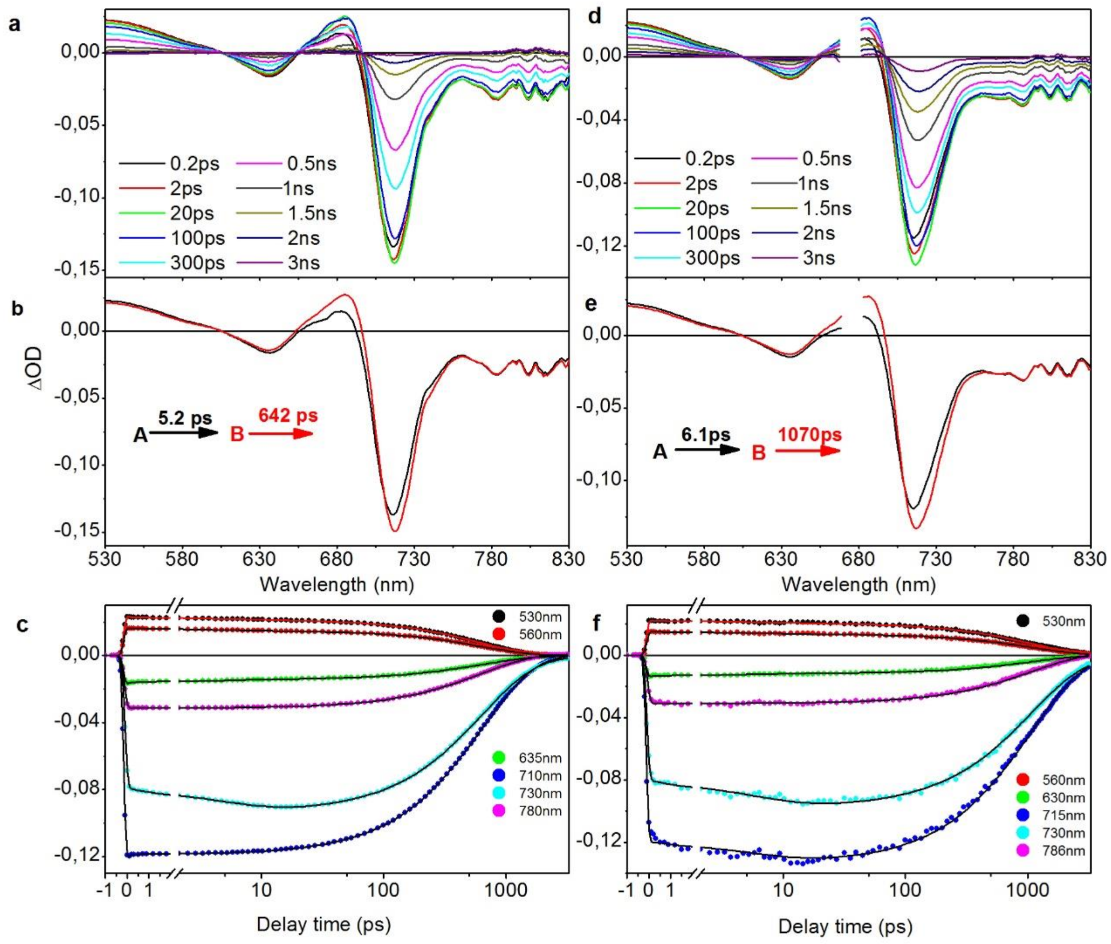

Figure S5. Transient absorption experimental results of iRFP720 and analyzed data in H_2O and D_2O. (a) Time resolved spectra in H_2O; (b) Global fitted EADS in H_2O; (c) Time decay traces in different wavelength and global fitted ones in H_2O; (d) Time resolved spectra in D_2O; (e) Global fitted EADS in D_2O; (f) Time decay traces in different wavelength and global fitted ones in D_2O.

SI4: TS1

NIR FP	Fluorescence lifetime (ps)	Transient absorption (ps)
iRFP702 – H_2O	749	692
iRFP702 – D_2O	1,349	1,030
iRFP713 – H_2O	676	607
iRFP713 – D_2O	1,200	1,015
iRFP720 – H_2O	636	642
iRFP720 – D_2O	1,200	1,070

TS1. Global fitted time constants for iRFP702, iRFP713 and iRFP720 proteins

SI5: Figure S6

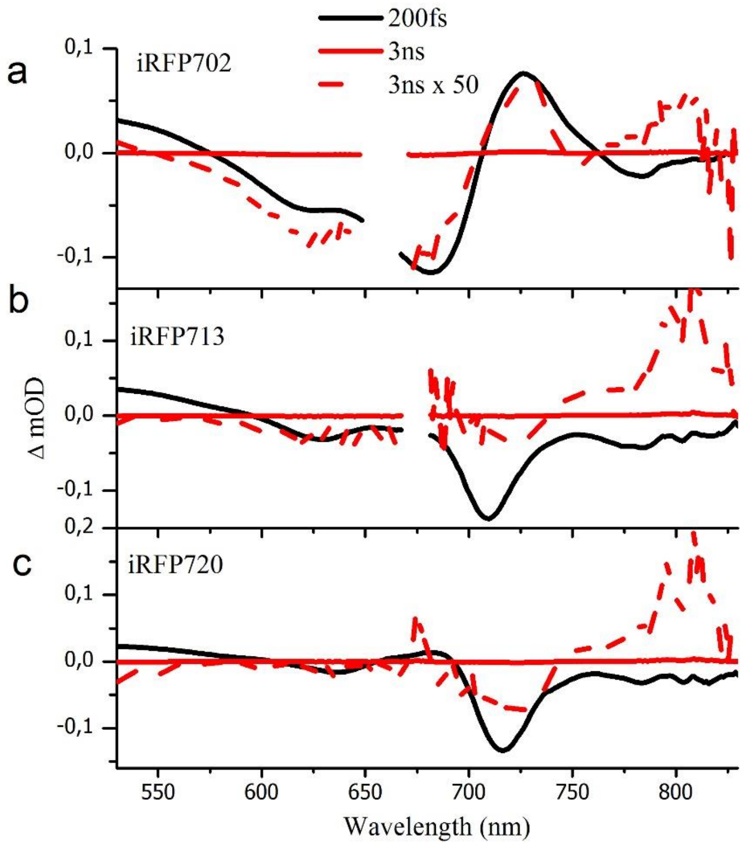

Figure S6. Time-resolved spectra taken at 200 fs (black) and 3 ns (red solid) and 3 ns expanded 50 times (red dash). The positive structures around 800 nm in the red dashed spectra results from instabilities of the white light continuum.

SI6: References

1 Zhu, J. Y. *et al.* Photoselective Ultrafast Investigation of Xanthorhodopsin and Its Carotenoid Antenna Salinixanthin. *Journal of Physical Chemistry B* **114**, 3038-3045 (2010).

2 Zhu, J. Y. *et al.* Photoionization and Electron Radical Recombination Dynamics in Photoactive Yellow Protein Investigated by Ultrafast Spectroscopy in the Visible and Near-Infrared Spectral Region. *Journal of Physical Chemistry B* **117**, 11042-11048 (2013).

3 Cohen, A. O. & Marcus, R. A. On the Slope of Free Energy Plots in Chemical Kinetics *The Journal of Physical Chemistry* **72**, 4249-4255 (1968).

4 Limbach, E. b. A. K. a. H.-H. The Kinetic Isotope Effect in the Photo-Dissociation Reaction of Excited-State Acids in Aqueous Solutions. *Isotope Effects In Chemistry and Biology* **Chapter 16** (2005).
